# Gender differential effect of college on political orientation over the last 40 years in the U.S.—A propensity score weighting approach

**DOI:** 10.1371/journal.pone.0279273

**Published:** 2023-01-18

**Authors:** Achim Edelmann, Stephen Vaisey

**Affiliations:** 1 médialab, Sciences Po, Paris, France; 2 Department of Sociology, Duke University, Durham, NC, United States of America; Chiang Mai University, THAILAND

## Abstract

It is well-known that the more educated people are, the more liberal views they tend to express. However, it is unclear whether this is due to college attendance itself or because those who go to college differ from those who do not in ways (directly or indirectly) related to their later political identification. In this paper, we therefore attempt to estimate the effect of college on political identification net of people’s tendencies to select into college using an inverse probability of treatment weighting approach. Based on data from the General Social Survey, we analyze how this effect has changed over time and whether college affects the political identification of women in the same ways as that of men. We find evidence consistent with the argument that college attendance politicizes both men and women. Moreover, we show that not only the general, but also the gender specific effects change markedly across the decades. This raises questions about the different mechanisms at play in how college mobilizes men and women politically.

## Introduction

The social sciences have long been interested in how education and socio-political orientation go together. It is well-known that the more educated people are, the more liberal views they tend to express. However, it is unclear whether this is due to college attendance itself or selection into college.

The observed college/non-college differences in political views might not only reflect generic influences of the college-going experience; college attendance as an experience has also likely changed over the decades. Furthermore, recent research suggests that effects of college experience depend strongly on personal and socio-demographic background. However, our understanding of such conditional effects is still limited. We neither know for sure whether college attendance *per se* has an effect on people’s socio-political orientations, nor whether such an effect is the same for different groups. For example, we don’t know whether the college experience yields the same effects for male and female students. This is particularly important as the gender balance in higher education has shifted over the past half-century to be predominantly female. If there is a difference in the average affect between men and women, then the average “effect of college” might be changing as well.

The goal of this paper is to contribute to a better understanding of these issues. We attempt to estimate the effect of college on political views (insofar as possible) net of selection. We further ask how this effect (if any) has changed over time and whether college has the same impact on the political identification of women as on that of men. In investigating these questions, our ultimate goal would be causal inference but that is likely not fully realistic. We can, however, use the data we have to estimate somewhat more realistic estimates of college effects.

To obtain nationally representative and historically consistent estimates of the college effect on political identification in the US, we draw on data from the General Social Survey. To attempt to estimate effects in observational data, we apply a counterfactual perspective that will help reduce confounding bias from selection [1]. Using covariate balancing propensity score weighting [2], we account for the possibility that those who go to college likely differ from those who do not in ways (directly or indirectly) related to their later political identification.

No strategy for causal inference guarantees identification with observational data. However, we find evidence consistent with the claim that attending college *does* influence people’s political identification but that it does so differently for men and women. We further show that not only the general, but also the gender-specific effects change markedly across the decades.

Our results have implications for understanding the effect of college on political identification in the US. Besides reconciling some contradictions in previous findings, these results underscore that scholars studying the relevance of college for people’s socio-political orientation would benefit from increased sensitivity to differential effects across groups and across time. This is especially crucial in light of the increasingly diverse background of college students and recent changes in the wider socio-political climate.

The rest of the paper proceeds as follows. We briefly review the research of how college effects students’ socio-political orientations. We highlight that, despite the existence of a great deal of research on this topic, many questions remain unsettled. Does attending college *per se* have an effect on people’s socio-political orientations beyond that of the broader, societal context? Are any such effects gender specific? We identify three methodological challenges in answering these questions. First, the ability to establish such a *net effect* in observational data where attending college is nonrandomly assigned; second, the need to model *effect heterogeneity*; and third, the need to account for possible changes of *societal influences* over time. After a short description of the data we use, we illustrate how we use covariate balancing propensity scores [2] to help address these methodological challenges. Summarizing our findings, we discuss the implications for the field and point towards further steps.

## Background

It is well-established that the more educated people are the more liberal the views they tend to express [3–9]. Equally well supported is that early adulthood is a period in life that is crucial to the formation of socio-political attitudes and that changes induced during that time are long-lasting, if not life-long [e.g. 5,6,10–13]. In particular, changes in students’ socio-political orientation during college were found to remain fairly stable over the life course, persisting even long into older age [e.g. 14–16].

However, the simplistic view of college as a homogenous cause for more liberal views has been criticized. From the 90s onwards, researchers have tried to understand the extent to which changes in students’ socio-political orientations were truly attributable to college attendance *per se*. Related works generally confirmed the association between higher education and socio-political attitudes and values (e.g., for political knowledge, activity, and participation, see [17–20]), but their results concerning the net effect of college were mixed. While some studies reported an increase in students’ liberalism during college (e.g. [21–23]), others found no effect of college on students’ political identification (e.g. [24]; more recently [25]), and evidence for only modest shifts towards more general liberal values after controlling for pre-college characteristics and other controls.

It has also been shown that the college effect on socio-political orientation is specific to cohorts [15,26,27]. For example, Cutler et al. [28] compare birth cohorts from the years 1894 till 1933, signaling greater liberalism for later cohorts during the years 1954 till 1972. Others indicated an increased political polarization as students going through college from 1984 till 1988 shifted from the middle of the spectrum towards both the left as well as the right (e.g. [24]; also [29,30]). Reflecting on these results, some argued that the observed shifts within students during college corresponds to shifts in the general population [30,31], thus indicating that the broader societal context could have as much of an impact on students as the college experience itself (also [24,32,33]; related [34]). Unfortunately, as Mayhew et al. [9] point out, studies employing methodologies appropriate to estimate net effects remain “scant” in this area of research.

More recently, scholars began to question whether the experience of college would be the same for everyone. Although studies since have emphasized that college experience might have different effects on different kinds of people, corresponding findings have remained inconclusive. This includes first attempts to study how the change in socio-political orientations experienced during college varies by gender (e.g. [35–39]). Summarizing the sparse findings, Pascarella et al. [7: 323ff] suggest that although women seem to enter college with more liberal attitudes than men, observed differences in how their socio-political orientations change during college disappear once other factors are accounted for; however, related insights are inconclusive and an understanding of such differential effects of college “remain[s] one of the unexplored frontiers” (also [40]).

As Hastie [41] points out, our lack of understanding is partly due to the available research being quite dated and not reflecting the more diverse background of recent college cohorts in term of age, ethnicity, and socio-demographic characteristics as well as life circumstances such as marriage status and part-time studies. Moreover, scholars showed that the student intake had changed markedly over time with an increasing number of conservatives now entering college [42].

In sum, to better understand the different effects that the college experience can have on different kinds of people, we need studies that include a wide variety of students (in terms of their non-traditional background), follow a research design that compares college graduates’ socio-political orientation with that of the general population, allows differentiating between students’ self-selection and socialization, and uses methods to capture conditional effects appropriately.

In this paper, we therefore aim to compare the effects of college experience on the political self-identification of women and men, focusing on corresponding trends across the last 40 years. Our goal is to attempt estimating (as well as possible given the limitations of our data) the effect of college on political views net of selection. While it is tempting to theorize the trends we find, we primarily see our work as contributing a rigorous empirical description of differences between men and women with and without college degrees. We hope this will lay the foundation for future work to develop a deeper understanding of the underlying mechanisms.

## Data

We approach these questions using the U.S. General Social Survey (GSS). Administered by the National Opinion Research Center (NORC), the GSS was fielded annually from 1972, and then biennially since 1994, to a representative sample of the adult American household population (excluding non-English speaking and institutionalized people). As a barometer of social attitudes and behavior, the GSS has consistently captured participants’ educational background and political self-identification, alongside other relevant socio-demographic variables, including characteristics of their upbringing. While some other surveys have collected more comprehensive data on participants’ background, none of them has done so consistently for representative samples as far back. The GSS is therefore a good choice for conducting representative comparisons between men and women across cohorts in the U.S..

## Measures

### College experience

The GSS asks participants to describe their educational background. We define college experience as having obtained a Bachelor’s or Master’s degree. (Note that this measure does not capture college drop outs, nor online degrees).

To avoid capturing participants in midst of a college experience, we exclude those aged 24 or less. To make our comparison as clear as possible, we want to compare those who attend and complete college with high school graduates who do not attend college. We therefore exclude participants with less than 12 years of education and those with some college experience who did not complete a bachelor’s degree.

### Political identification

To measure political identification, respondents were shown a seven-point scale, displaying political views—“extremely liberal” (1), “liberal” (2), “slightly liberal” (3), “moderate” (4), “slightly conservative” (5), “conservative” (6), and “extremely conservative” (7)—and asked: “Where would you place yourself on this scale?” Since this question was first asked in 1974, our analysis will be limited to waves 1974 to 2018.

### Covariates

We include several demographic and socio-economic variables known to affect college attendance. In particular, we model college attendance based on *sex* (1 = male), *age* (range = 25–89 or older), and whether respondents identified as *white* (1 = white) (on the use of sex as an indicator for gender, see note 1 in S1 Notes).

We account for *father’s occupation* based on the 10-fold census occupation codes and for *father’s occupational prestige* based on the 10-fold 2010 census occupation classification. To capture differences within these coarse categories, we account for the *mean deviation* of prestige scores within occupational categories. We also account for *father’s* and *mother’s highest educational degree* obtained as measured on a 5-point scale.

In addition to this, we include several variables that describe the conditions in which respondents grew up. In particular, we use a set of questions that asked participants to describe characteristics of their upbringing at the age of 16 and thus *prior* to their eventual college experience. This includes: the *family arrangement* (4 categories: lived with “father”, “mother”, “both”, or in “other arrangement”); whether the *father was self-employed* or worked for someone else; the *religion* raised in (“Protestant”, “Catholic”, “Jewish”, “Buddhism”, “Hinduism”, “Moslem/Islam”, “Orthodox-Christian”, “Christian”, “None”, and “Other” in which we also collapse “Other eastern”, “Native American”, and “Inter-nondenominational” due to small cell sizes); a 7-fold categorization of the *denomination* raised in; the *state* or foreign country (10 categories); and the *type of place* they lived in at the age of 16 (6 categories, ranging from “country, non-farm” to “city greater than 250,000”).

For any of the categorical covariates, we treat missing data resulting from question filtering as a valid value. For the mean deviation of father’s prestige score, we encode such missings as zeros. We do this to ensure that subgroups that might be characteristic for college or non-college goers (such as respondents who grew up without a father) are not lost in our analysis.

To capture period effects, we include the *year* the survey was fielded. Moreover, we include a categorical variable for *cohort decades* and terms for its interactions with all of the above variables.

## Analytic approach

### College as non-random assignment

To provide credible estimates of the effect of college on political identification, we need to address the fact that college attendance is nonrandomly assigned. The classic strategy to estimate the effect of college has been to include it alongside with other covariates in a regression. This approach requires correctly specifying the functional relationship between the covariates and the outcome, which is difficult given how underdeveloped our theoretical accounts are of the process generating the outcome.

Of course, we know that selection into college is not random but dependents on a wide variety of factors. Those who go to college differ systematically from those who don’t in characteristics that might be (directly or indirectly) related to their political self-identification after college. Directly comparing both groups therefore does not yield an unbiased estimate of the college effect.

### Inverse probability of treatment weighting

To move a step closer to estimating the effects of the college experience on political identification with observational data, we use a semi-parametric approach to inverse probability of treatment weighting (IPTW). This gives us a better chance to isolate causal factors by removing biases associated with differences between participants who go to college and those who don’t. Unlike regression, our approach does not require correctly specifying the functional relationship between these background characteristics and the outcome. Instead, it simply weights the data such that the treatment and control groups have nearly identical univariate distributions on all observed covariates.

IPTW involves two steps: First, we determine the probability for each participant to go to college. Second, in comparing the political views of respondents who went to college with those who did not, we weight each control case such that *weight_i_* = *p_i_*/(1−*p_i_*), where *p* is the estimated probability of going to college (for our use of survey weights, see note 2 in S1 Notes). This approach gives more weight to respondents who “look like” college graduates and less weight to respondents who do not. As a result, we effectively compare two (weighted) groups that are much more similar in their pre-treatment characteristics (on IPTW’s efficiency and validity in estimating average treatment effects, see note 3 in S1 Notes).

Specifying the set of covariates to include requires care. First, to prevent conditioning on possible colliders (and to not underestimate the effect of college in step two) we can only use variables that describe pre-college conditions [1]. Luckily, the GSS asked participants about their parents’ socio-economic background (including their occupation, income and education) as well as about major characteristics of their upbringing at the age of 16 (including their family arrangement, religious denomination, and residential area and type).

Second, ensuring the fit of a propensity score model requires checking the covariate balance among the treated and untreated and, if necessary, adjusting the model accordingly. To avoid biases from ad hoc specification search [43], we use the covariate balancing propensity score (CBPS) method [2] (for further developments/improvements for CBPS, see [44]). Unlike traditional propensity score modeling, which typically uses a logit model to estimate treatment probability (and thus depends on functional form assumptions), CBPS models treatment assignment probability while simultaneously optimizing covariate balance using an empirical likelihood approach. This has the advantage of reducing biases induced by manual balance checks and related model adjustments. CBPS shows desirable qualities and outperforms other semi-parametric strategies, including genetic matching and entropy matching, in simulation studies [45,46].

### Analytical strategy

To explore the gender differential effect of college on participants’ political views, we proceed in two steps. We first ask whether college politicizes participants *per se* as signaled by political views other than “moderate” at the middle of the spectrum. To explore gender differential effects, we analyze mean differences between women and men depending on whether they went to college or not.

In a second step, we ask what kind of political view college induces. To explore this, we focus on participants who identified as (to any degree) liberal or conservative and analyze their proportions among those who went to college and those who did not.

In both of these steps, we plot respective mean differences over time and contrast observational results with causal results approximated by the propensity score weighting approach.

### Analytical sample

We focus on cohorts from 1900 to 1989, exclude cases in the black oversample from waves 1982 and 1987, and drop cases with missing data (other than the ones explicitly encoded) on any of the described covariates. The resulting sample for the matching consists of 35,690 respondents. (For the sample distribution by survey year, cohort, and gender, see Tables 1 and 2 in S1 Table; for further proportionate distributions of the sample by survey year, see S1 File).

## Results

### Predicting going to college

We begin by estimating the probability for each participant to go to college using CBPS. We focus on characteristics that capture factors potentially at work prior to the decision to attend college and that were asked across all years in the GSS.

In particular, we seek college/non-college balance on participants’ *age* (continuous), survey year (continuous), *sex*, *white racial identity*, *father’s occupational prestige*, *mean deviation of father’s prestige* within their (10-fold) occupational category, *father’s* and *mother’s highest educational degree*, and a series of covariates that describe the participants’ living arrangement at age 16, including their *family arrangement*, *religion* and *religious denomination*, *region*, *state*, and *type of place* lived in, and whether the father was *self-employed*. Finally we add dummies for *cohort decades* and their interactions with any of the variables in the model; note, this includes the interaction between *sex* and *cohort decades* to account for the possibility that for women from different cohorts going to college/the college experience means different things.

**Fig 1:**
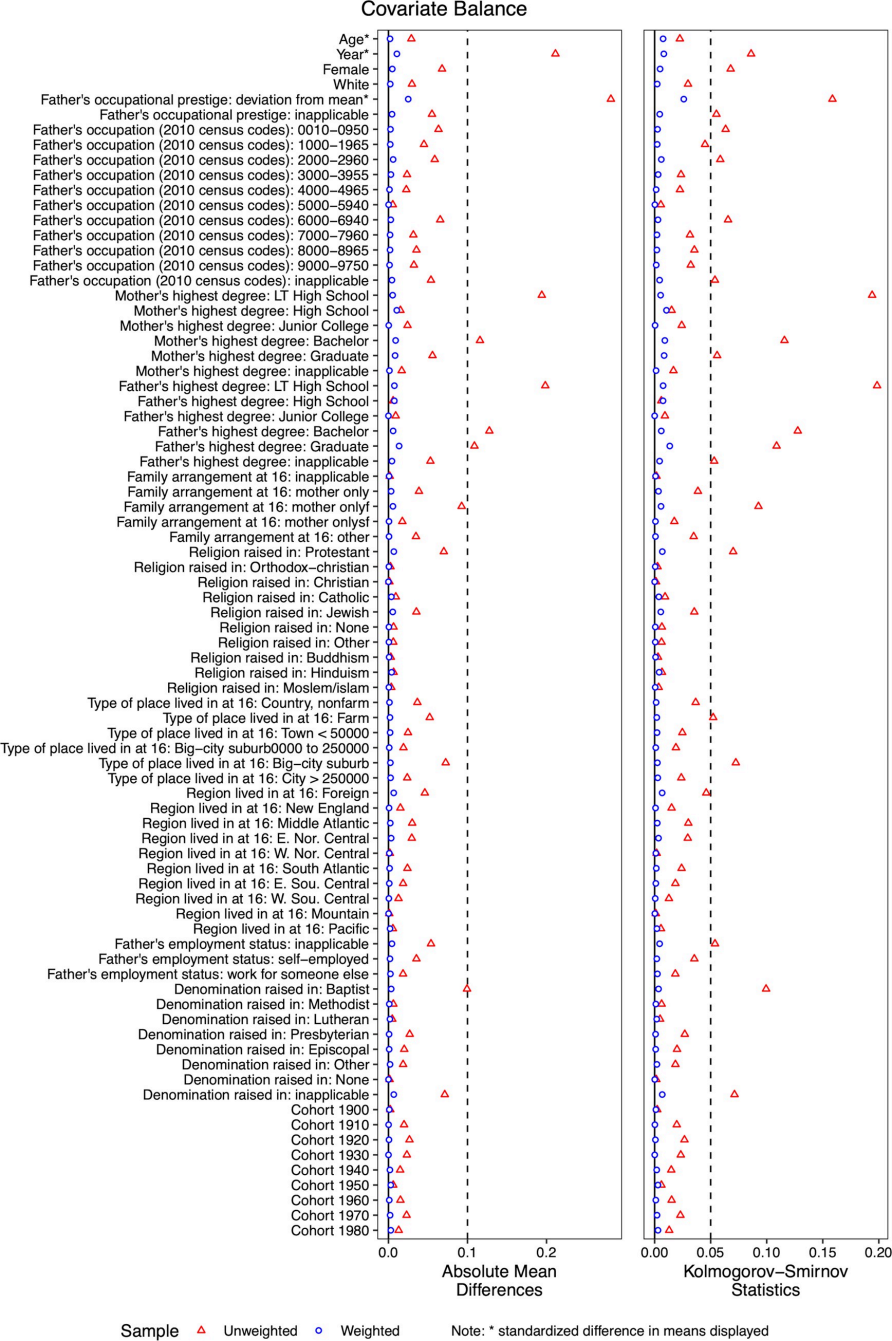
Covariate balance plot unadjusted versus adjusted mean difference and Kolmogorov-Smirnov statistic. Horizontal lines mark critical values 0.1 and 0.05 respectively.

Fig 1 displays the absolute mean difference and Kolmogorov-Smirnov statistics for the weighted and unweighted case. As shown, the CBPS weights substantially improve balance across all variables

### Does college politicize men and women?

We begin by exploring whether college politicizes participants. We focus on the proportion of participants that signaled political views other than “moderate” and plot mean differences between those who went to college from those who did not.

**Fig 2:**
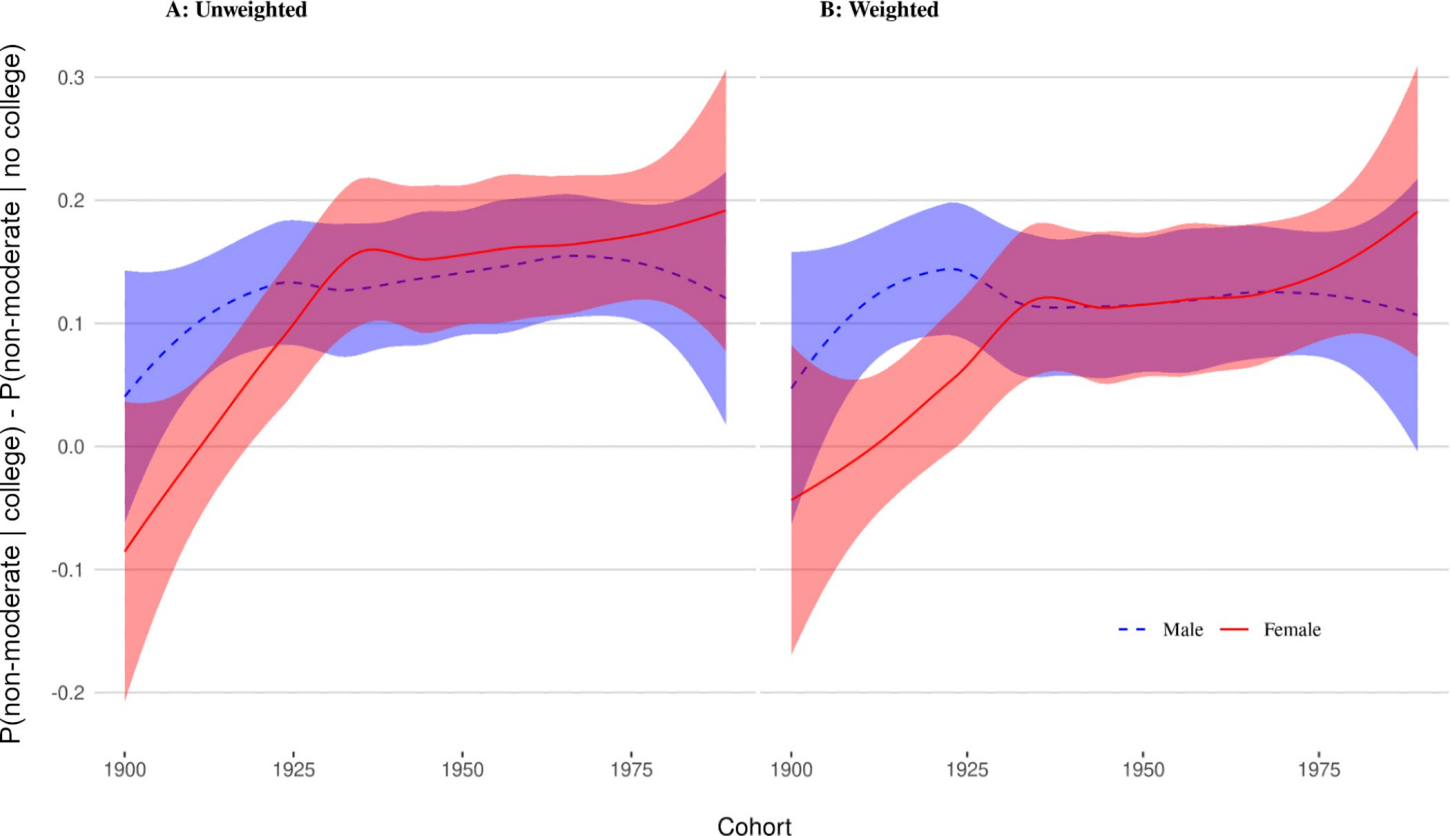
Difference in proportion of non-moderates between college goers and non-goers by gender.

Fig 2 shows the (smoothed) trend of respective mean differences across cohorts for men and women. It shows that going to college has politicized both men and women as reflected in the proportion of those who signal political views other than “moderate” among college goers compared to non-goers. If we focus on mere observational comparisons, among men, this politicization effect reached a more or less stable level of about 13 percentage points with cohorts in the early 1920s, peaking at about 15 points for the late 1960s, and showing a slight drop for cohorts after 1970s. For women, the politicization effect was even negative for the cohorts prior WWI (for which data is sparse) but caught up with that of men and even outpacing it by about 1 to 3 points in the early 1930s. It then continues to rise slowly to about 19 points for the 1989 cohort.

The CBPS-weighted comparisons show minor changes compared to the unweighted data. For both men and women, causal estimates of the polarization effect are slightly less pronounced, meaning selection is clearly playing some role. We notice, however, that for the cohorts prior the early 1930s, differences between men and women do increase slightly, signaling that while college already in the early years had a strong politicizing effect for men, this wasn’t the case for women. For cohorts after the early 1930s, a causal perspective reduces the observed differences between men and women. The politicization effect of college is basically indistinguishable for men and women, settling on about 12 percentage-point increase in having a political position. This signals that any observed differences for those cohorts likely reflect tendencies of self-selection.

For the later cohorts, however, the male and female estimates diverge. Since the early 1970s, college seems to have an increasing relationship to men’s politicization and a decreasing relationship to women’s politicization. We explore this in the next section.

### How does college politicize men and women?

**Fig 3:**
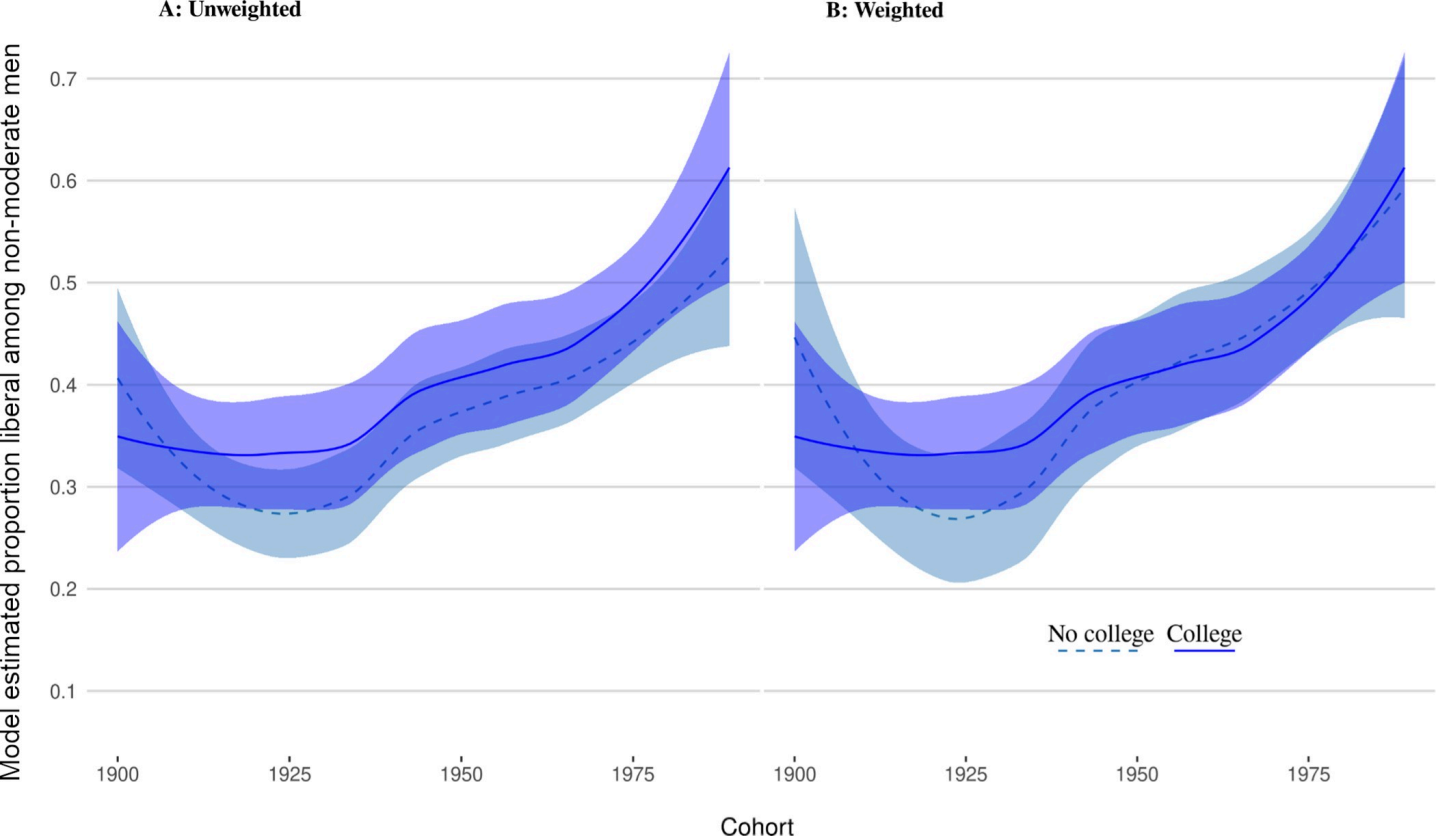
Men’s proportion of liberals among non-moderates by college attendance.

**Fig 4:**
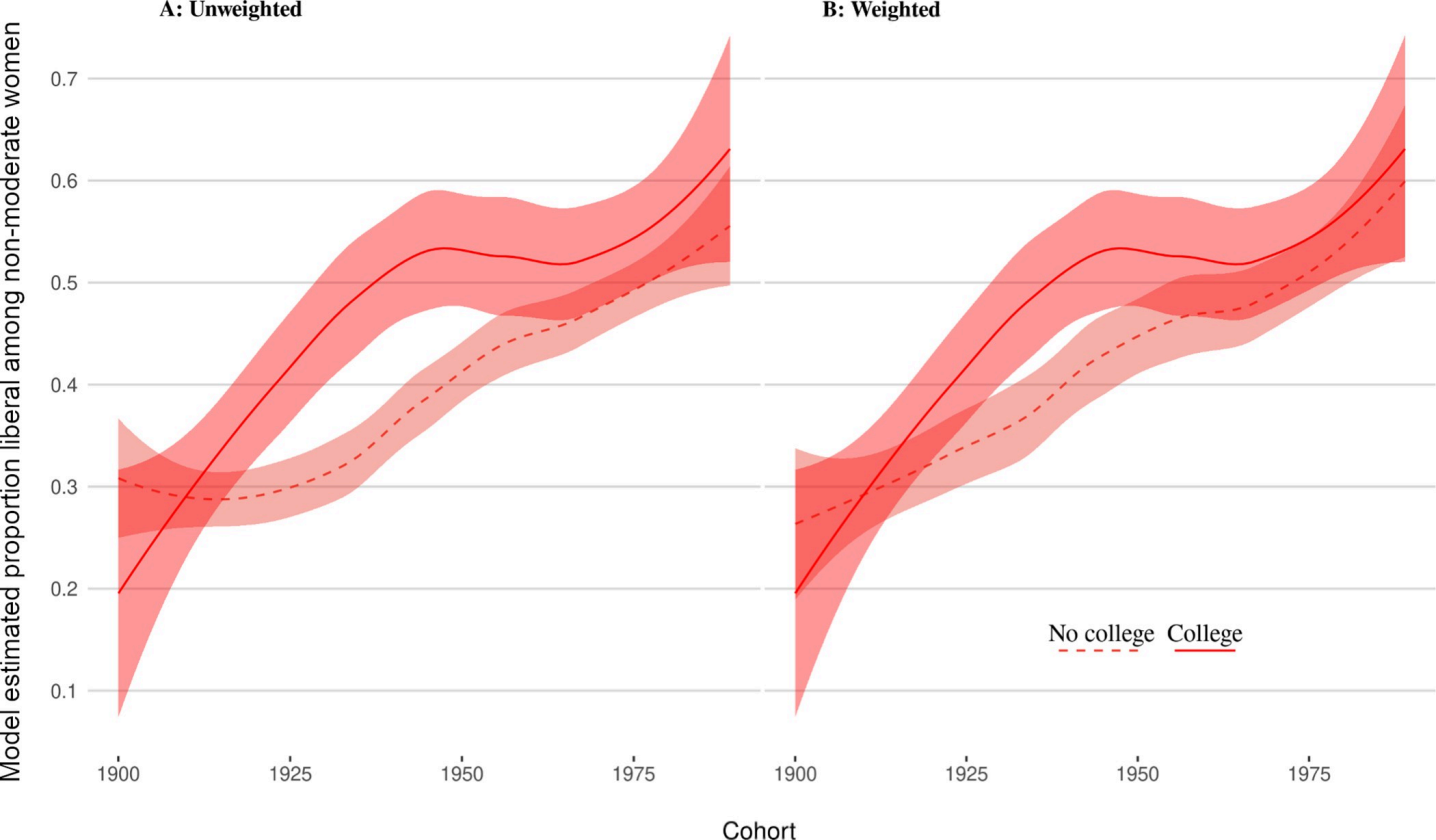
Women’s proportion of liberals among non-moderates by college attendance.

The above shows that for several decades of cohorts attending college politicized men and women to a similar degree. However, this does not imply that college politicized men and women in the same way. To explore this issue, we focus on those who did not identify as “moderates” and analyze the proportion of liberals among those. Figs 3 and 4 show the proportions of liberals among non-moderates for men and women broken down by college attendance.

Here the differences between the solid and dashed lines are estimates of the “treatment.” For men, the unweighted estimates (panel A) suggest a fairly consistent liberalization bump of about 4–5 percentage points. When we weight the samples using CBPS (panel B), however, the two lines become almost identical. This suggests that although college might *politicize* men it does not appear to make them more likely to be liberal or conservative.

For women, the pattern is different. Here the weighted and unweighted comparisons are very similar. The story they tell is of large increases in liberalization for early GSS cohorts (approximately 1910 to 1960) but declining effects for more recent cohorts. This suggests that college may have once played a much stronger liberalizing role for women in the past than it does today.

## Discussion and conclusion

In this paper, we attempted to estimate the effect of college attendance on men’s and women’s political self-identification net of their tendencies to self-select into college. Our analysis yielded several findings. First, we found evidence for a politicization effect of college as reflected in lower rates of moderates among college goers compared to non-goers. Already for early cohorts, this effect was strong for men and less pronounced for women. For cohorts from the 1930s to the early 1970s, this politicization effect remained stable and indistinguishable between men and women; related effect differences that can be observed between cohorts of men and women from that time likely reflect tendencies of self-selection. For later cohorts, however, college indeed seems to have politically mobilized women more than men.

Second, our results clearly reveal that college has not mobilized men and women in the same way. Rates of liberals increased for both throughout the decades. While we notice a mild liberalization effect for earlier cohorts of men, any observable differences for cohorts after 1944 seem to be due to tendencies of self-selection. This suggests that, for men from that time, college might have functioned as an amplifier of political identification, enabling or stimulating them to take sides on either side of the political spectrum.

For women, we find a strong liberalization effect among the early cohorts, peaking with the 1933 cohort at a level about twice as high as that for men. This effect declines starting with the 1944 cohort, settling on a smaller but stable level for the 1960s and later cohorts. Unlike for men, for women observed differences in the proportion of liberals between college goers and non-goers cannot be explained by self-selection. One possible interpretation of this could be that the college experience might not only enable or encourage women to take a political position *per se* but (possibly mediated through peer and faculty effects) also exposes them to social roles and ideas that are more liberal than would have experienced otherwise.

This study is of course limited. For one, as with most institutions, also attending college is a highly gendered experience in which roles, behaviors, and identities of their members reflect a socially constructed spectrum that goes beyond a simple dichotomy of male/female. While capturing an influential dimension of gender, participants’ sex should only be seen as an indicator of this differentiation. Moreover, our use of covariate-balancing propensity score weighting is useful but cannot remove selection bias that works through channels unrelated to the measured covariates. Although we have taken great care in selecting covariates that could be related to people’s selection into college but not affected by the college experience themselves—including their father’s occupation and occupational prestige, their parents’ highest education, and the place, religion, and denomination they were raised in prior to college—we therefore cannot rule out self-selection that works through channels unrelated to these covariates.

Despite these limitations, this study has important implications for research and public policy. For research aiming to understand the effect of college on political identification in the U.S., it helps explain why previous studies have found mixed effects of college on political identification. Differences in estimates might simply reflect aspects of the historic trend we observe and/or tendencies of self-selection that are left unaccounted for. This especially concerns studies that have found no differences in the effect of college attendance on women and men. In doing so, our study also demonstrates that a deeper understanding of how college affects people’s political identification requires methodological approaches that are sensitive to differential effects across groups and across time.

For public policy more broadly, this study warns against naive generalizations and simplifications when considering social institutions. On the one hand, the results of this study demonstrate that the seeming historic continuity of institutions such as college might well mask substantial changes in the kind of people they attract and the impact they have on them. Policy makers should therefore be careful when extrapolating related knowledge from the past to the present. On the other hand, this study cautions against viewing college as a uniform and overall equalizing experience. As the results show, going to college does not affect everyone in the same way and can even amplify existing (political) differences among some.

## Supporting information

S1 Table (PDF)

S1 Notes (PDF)

S1 File Supplementary descriptives. (PDF)

## References

[1] Morgan SL, Winship C. Counterfactuals and causal inference. Cambridge University Press; 2015.

[2] Imai K, Ratkovic M. Covariate balancing propensity score. Journal of the Royal Statistical Society: Series B (Statistical Methodology). 2014 Jan; 76(1): 243–63.

[3] Feldman KA, Newcomb TM. The Impact of College on Students: An Analysis of Four Decades of Research. San Francisco, CA: Jossey-Bass; 1973.

[4] Stephens WN, Long CS. Education and political behavior. Political science annual. 1970; 2(1): 3–33.

[5] Hyman HH, Wright CR. Education’s lasting influence on values. Chicago: University of Chicago Press. 1979.

[6] Pascarella ET, Terenzini PT. How College Affects Students: Findings and Insights From Twenty Years of Research. San Francisco, CA: Jossey-Bass; 1991.

[7] Pascarella ET, Terenzini PT. How College Affects Students: A Third Decade of Research. Volume 2. Jossey-Bass, Indianapolis, IN; 2005.

[8] Kingston PW, Hubbard R, Lapp B, Schroeder P, Wilson J. Why education matters. Sociology of Education. 2003 Jan 1: 53–70.

[9] Mayhew MJ, Rockenbach AB, Bowman NA, Seifert TA, Wolniak GC, Pascarella ET, et al. How college affects students: 21st century evidence that higher education works. San Francisco, CA: Jossey-Bass. 2016.

[10] Alwin DF, Hauser RM, Sewell WH. Colleges and achievement. In: Sewell WH, Hauser RM. Education, Occupation, and Earnings. Achievement in the Early Career. New York: Academic Press; 1975. p. 113–42.

[11] Fendrich JM. Activists Ten Years Later: A Test of Generational Unit Continuity 1. Journal of Social Issues. 1974 Jul; 30(3): 95–118.

[12] Krosnick JA, Alwin DF. Aging and susceptibility to attitude change. Journal of Personality and Social Psychology. 1989 Sep; 57(3): 416. doi: 10.1037//0022-3514.57.3.416 2778632

[13] Kiley K, Vaisey S. Measuring stability and change in personal culture using panel data. American Sociological Review. 2020 Jun; 85(3): 477–506.

[14] Newcomb TM., Koenig KE, Flacks R, Warwick DP. Persistence and Change. Persistence and Change: Bennington College and Its Students After Twenty-Five Years. New York: John Wiley & Sons; 1967.

[15] Alwin DF, Cohen RL, Newcomb TM. Political attitudes over the life span: The Bennington women after fifty years. Univ of Wisconsin Press; 1991.

[16] Brown D., Pacini R. The Vassar Classes of 1957 and 1958: The ideal student study. In: Hulbert KD, Schuster DT. Women’s lives through time: Educated American women of the twentieth century. Jossey-Bass/Wiley; 1993. p. 93–116.

[17] Tuma J, Geis S, Carroll CD. High school and beyond: 1992 descriptive summary of 1980 high school sophomores 12 years later. National Center for Education Statistics. Washington, DC: US Government Printing Office Nr. 95–304; 1995.

[18] Nie NH, Junn J, Stehlik-Barry K. Education and democratic citizenship in America. University of Chicago Press; 1996 Nov 15.

[19] Nolin MJ, Chapman C. Adult Civic Involvement in the United States. National Center for Education Statistics. Washington, DC: US Government Printing Office. Nr. 97–906; 1997.

[20] Berkner L, He S, Cataldi EF. Descriptive summary of 1995–96 beginning postsecondary students: Six years later. NCES. 2003; 151: 61.

[21] Lottes IL, Kuriloff PJ. The impact of college experience on political and social attitudes. Sex Roles. 1994 Jul; 31(1): 31–54.

[22] Underwood L, Maes B, Alstadt L, Boivin M. Evaluating changes in social attitudes, character traits, and liberal-arts abilities during a four-year program at a Christian college. Research on Christian Higher Education. 1996; 3: 115–28.

[23] Loeb RC, Magee PM. Changes in attitudes and self-perceptions during the first two years of college. Journal of College Student Development. 1992 Jul; 33(4): 348–355.

[24] Astin AW. What matters in college? Four critical years revisited. San Francisco, CA: Jossey-Bass. 1993.

[25] Campbell C, Horowitz J. Does college influence sociopolitical attitudes? Sociology of Education. 2016 Jan; 89(1): 40–58.

[26] Alwin DF, Krosnick JA. Aging, cohorts, and the stability of sociopolitical orientations over the life span. American journal of sociology. 1991 Jul 1; 97(1): 169–95.

[27] Newcomb TM. Personality and Social Change; Attitude Formation in A Student Community. New York: Dreyden Press; 1943/1957.

[28] Cutler SJ, Kaufman RL. Cohort changes in political attitudes: tolerance of ideological non conformity. Public Opinion Quarterly. 1975 Jan 1; 39(1): 69–81.

[29] Schiff TW. Political identification and political attitudes of American college students. University of California, Los Angeles; 1993.

[30] Dey EL. Undergraduate political attitudes: An examination of peer, faculty, and social influences. Research in Higher Education. 1996 Oct; 37(5): 535–54.

[31] Dey EL. Undergraduate political attitudes: Peer influence in changing social contexts. The Journal of Higher Education. 1997 Jul 1; 68(4): 398–413.

[32] Phelan J, Link BG, Stueve A, Moore RE. Education, social liberalism, and economic conservatism: Attitudes toward homeless people. American Sociological Review. 1995 Feb 1: 126–40.

[33] Mariani MD, Hewitt GJ. Indoctrination U.? Faculty ideology and changes in student political orientation. PS: Political Science & Politics. 2008 Oct; 41(4): 773–83.

[34] Kam CD, Palmer CL. Reconsidering the effects of education on political participation. The Journal of Politics. 2008 Jul; 70(3): 612–31.

[35] Lottes IL, Kuriloff PJ. Sexual socialization differences by gender, Greek membership, ethnicity, and religious background. Psychology of Women Quarterly. 1994 Jun; 18(2): 203–19.

[36] Knox WE. Does College Make a Difference? Long-Term Changes in Activities and Attitudes. Contributions to the Study of Education, Number 59. Greenwood Publishing Group, Westport, CT; 1993.

[37] Martin RJ, Koppelman K. The Impact of a Human Relations/Multicultural Education Course on the Attitudes of Prospective Teachers. Journal of Intergroup Relations. 1991; 18(1): 16–27.

[38] Smith DG, Morrison DE, Wolf LE. College as a gendered experience: An empirical analysis using multiple lenses. The Journal of Higher Education. 1994 Nov 1; 65(6): 696–725.

[39] Sax LJ. The gender gap in college: Maximizing the developmental potential of women and men. San Francisco, CA: Jossey-Bass; 2008.

[40] Pascarella ET. How college affects students: Ten directions for future research. Journal of College Student Development. 2006; 47(5): 508–20.

[41] Hastie B. Higher education and sociopolitical orientation: The role of social influence in the liberalisation of students. European Journal of Psychology of Education. 2007 Sep; 22(3): 259–74.

[42] Pryor JH, Hurtado S, Saenz VB, Santos JL, Korn WS. The American Freshman: Forty Year Trends. Los Angeles: Higher Education Research Institute. 2007.

[43] Diamond A, Sekhon JS. Genetic matching for estimating causal effects: A general multivariate matching method for achieving balance in observational studies. Review of Economics and Statistics. 2013 Jul 1; 95(3): 932–45.

[44] Fan J, Imai K, Liu H, Ning Y, Yang X. Improving covariate balancing propensity score: A doubly robust and efficient approach. Technical Report, Princeton University. https://imai.fas.harvard.edu/research/CBPStheory.html; 2016.

[45] Frölich M, Huber M, Wiesenfarth M. The finite sample performance of semi-and non-parametric estimators for treatment effects and policy evaluation. Computational Statistics & Data Analysis. 2017 Nov 1; 115: 91–102.

[46] Wyss R, Ellis AR, Brookhart MA, Girman CJ, Jonsson Funk M, LoCasale R, et al. The role of prediction modeling in propensity score estimation: an evaluation of logistic regression, bCART, and the covariate-balancing propensity score. American journal of epidemiology. 2014 Sep 15; 180(6): 645–55. doi: 10.1093/aje/kwu181 25143475 PMC4157700

