# Supplementary material for: Gender differential effect of college on political orientation over the last 40 years in the U.S.—A propensity score weighting approach
Source: PLoS One. 2023 Jan 18;18(1):e0279273. doi: 10.1371/journal.pone.0279273 (PMC9847899; doi:10.1371/journal.pone.0279273)
Supplement: S1 Table — (PDF) [file pone.0279273.s001.pdf]

S1 Tables.

Table 1. Sample distribution by survey year.

year	N
1974	658
1975	667
1976	676
1977	708
1978	767
1980	753
1982	770
1983	468
1984	791
1985	888
1986	857
1987	829
1988	876
1989	923
1990	859
1991	902
1993	1036
1994	2053
1996	1890
1998	1851
2000	1812
2002	939
2004	936
2006	3263
2008	1388
2010	1420
2012	1347
2014	1821
2016	1970
2018	1572

Table 2. Sample distribution by cohort and gender.

cohort	Men	Women	cohort	Men	Women
1900	7	13	1945	298	344
1901	10	14	1946	333	410
1902	10	30	1947	428	517
1903	8	18	1948	456	475
1904	15	25	1949	401	439
1905	22	18	1950	422	455
1906	20	19	1951	395	470
1907	19	31	1952	414	483
1908	20	45	1953	390	472
1909	33	51	1954	352	429
1910	29	40	1955	394	428
1911	40	64	1956	388	468
1912	39	67	1957	366	491
1913	36	76	1958	400	502
1914	49	92	1959	365	462
1915	57	88	1960	363	426
1916	79	99	1961	366	363
1917	65	119	1962	308	402
1918	91	122	1963	311	370
1919	83	121	1964	291	359
1920	83	118	1965	289	317
1921	112	145	1966	251	271
1922	100	143	1967	230	278
1923	127	157	1968	236	287
1924	115	167	1969	213	288
1925	114	174	1970	220	264
1926	118	182	1971	181	244
1927	139	169	1972	171	196
1928	141	163	1973	160	216
1929	133	182	1974	154	199
1930	142	203	1975	160	179
1931	131	176	1976	118	159
1932	149	198	1977	133	171
1933	149	182	1978	102	143
1934	173	230	1979	146	154
1935	185	224	1980	109	170
1936	195	248	1981	116	154
1937	185	228	1982	101	114
1938	202	228	1983	81	107
1939	198	239	1984	89	104
1940	216	285	1985	78	110
1941	257	319	1986	59	68
1942	259	333	1987	64	70
1943	309	349	1988	47	70
1944	302	364	1989	58	61
