# Supplementary material for: Gender differential effect of college on political orientation over the last 40 years in the U.S.—A propensity score weighting approach
Source: PLoS One. 2023 Jan 18;18(1):e0279273. doi: 10.1371/journal.pone.0279273 (PMC9847899; doi:10.1371/journal.pone.0279273)
Supplement: S1 Notes — (PDF) [file pone.0279273.s002.pdf]

S1 Notes.

1. The concept of theoretical relevance is gender, but the GSS only holds information on participants' sex. Albeit an imperfect solution, we therefore take sex as an indicator of gender. We thank an anonymous reviewer for raising this point.

2. In both steps, we weigh each case with the GSS population survey weights (WTSSALL) to account for number of adults in the household and subsampling of non-respondents after 2004.

3. IPTW has been shown to remove selection biases that work through factors associated with the controls, yielding efficient estimates of the average treatment effects [1]. Moreover, unlike propensity score matching which defines particular cases from both groups to compare, IPTW preserves external validity and ensures that all respondents in the sample can be used.

References

1. Hirano K, Imbens GW, Ridder G. Efficient estimation of average treatment effects using the estimated propensity score. Econometrica. 2003 Jul; 71(4): 1161-89
