# Supplementary material for: Gender differential effect of college on political orientation over the last 40 years in the U.S.—A propensity score weighting approach
Source: PLoS One. 2023 Jan 18;18(1):e0279273. doi: 10.1371/journal.pone.0279273 (PMC9847899; doi:10.1371/journal.pone.0279273)

S1 Supplementary descriptives.

College experience

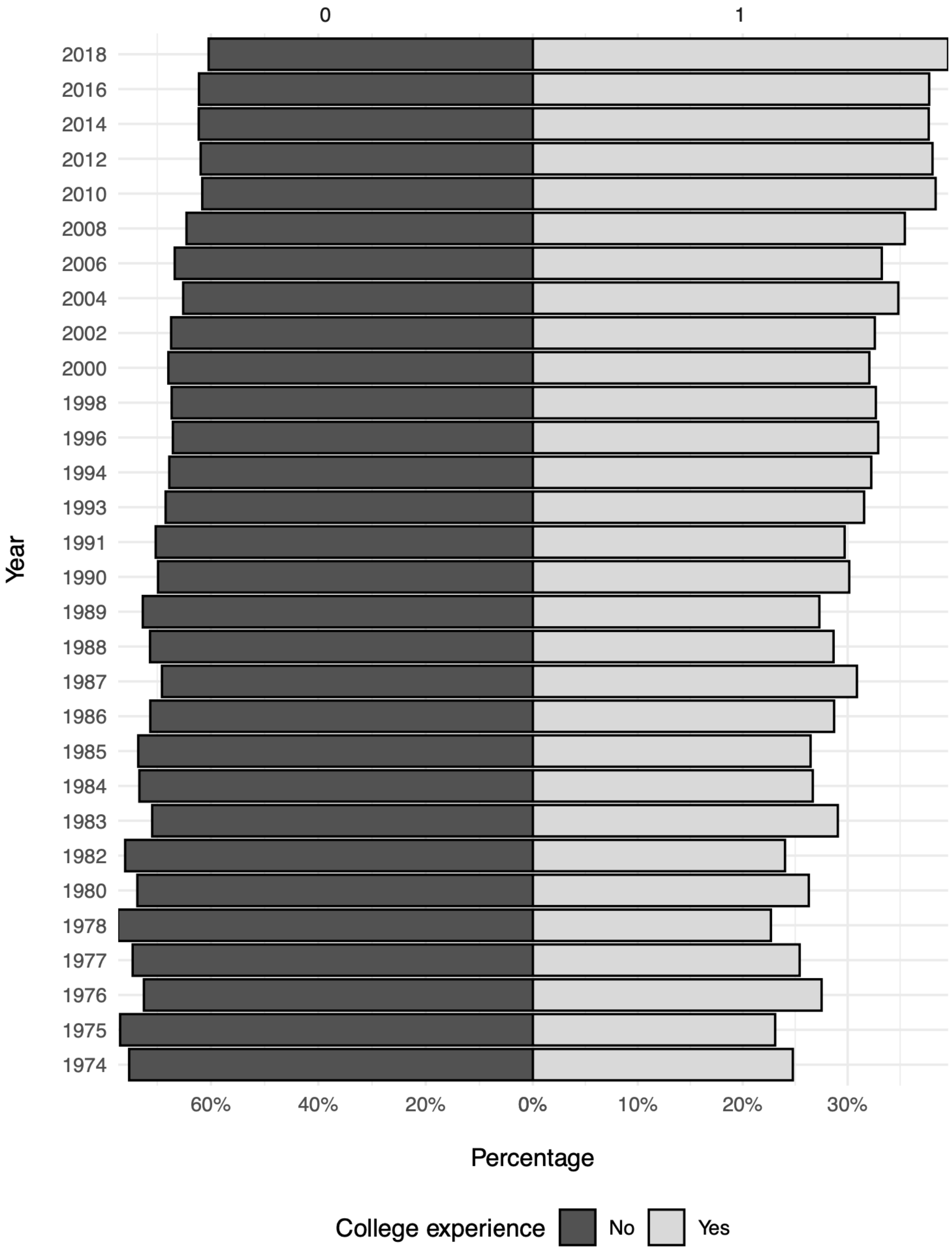

Political identification

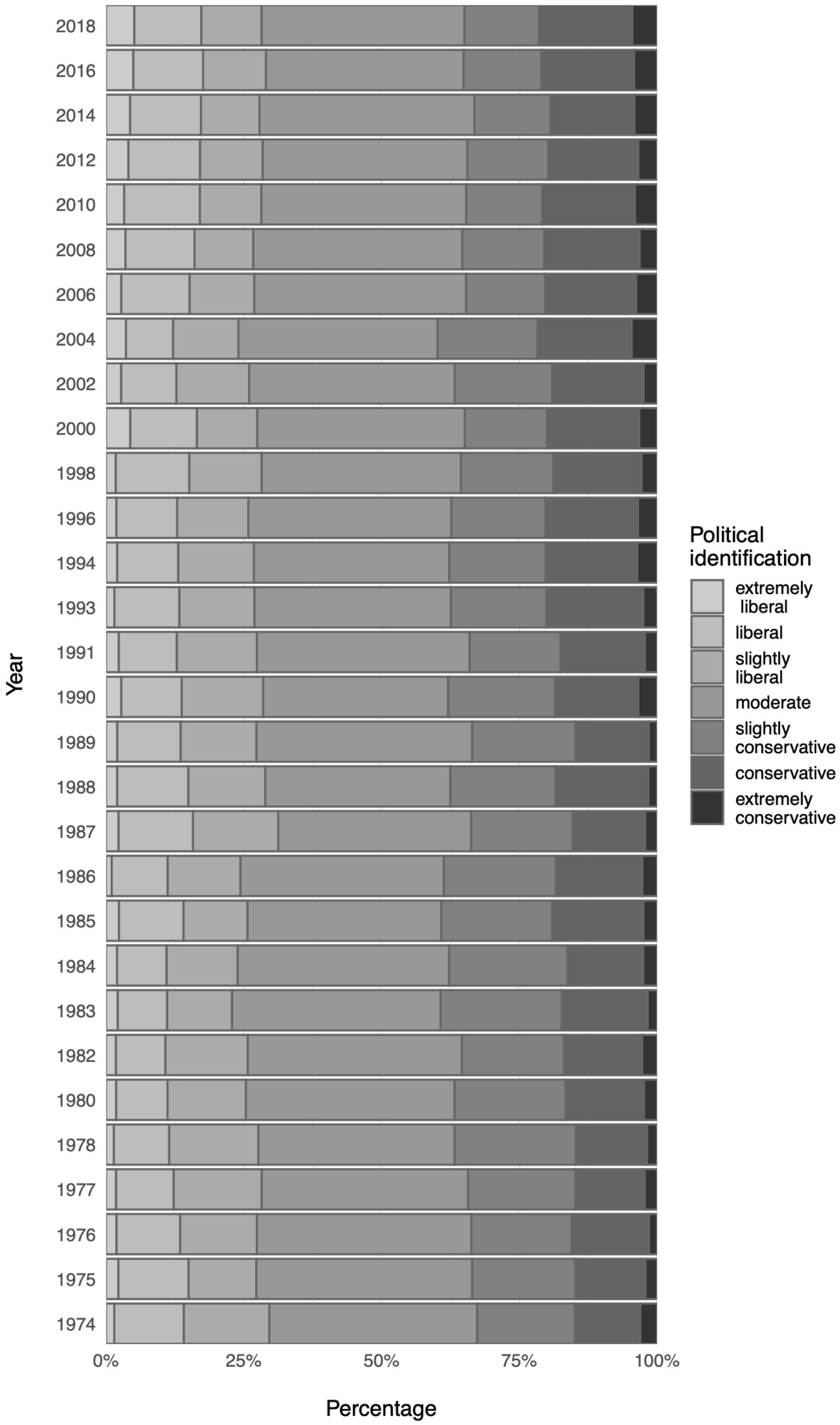

Sex

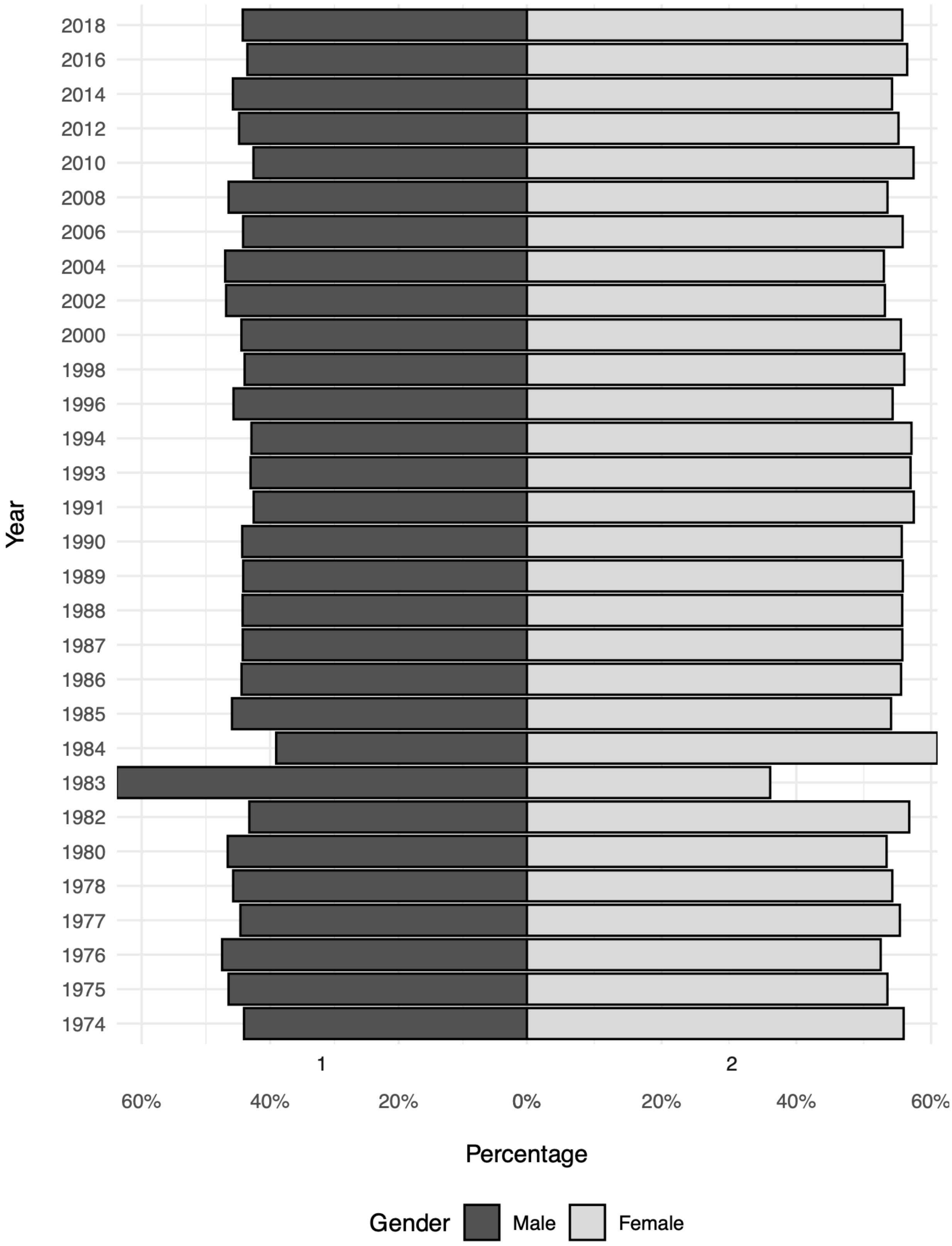

Year
2018
2016
2014
2012
2010
2008
2006
2004
2002
2000
1998
1996
1994
1993
1991
1990
1989
1988
1987
1986
1985
1984
1983
1982
1980
1978
1977
1976
1975
1974
1
2
60%
40%
20%
0%
20%
40%
60%
Percentage
Gender
Male
Female

Age

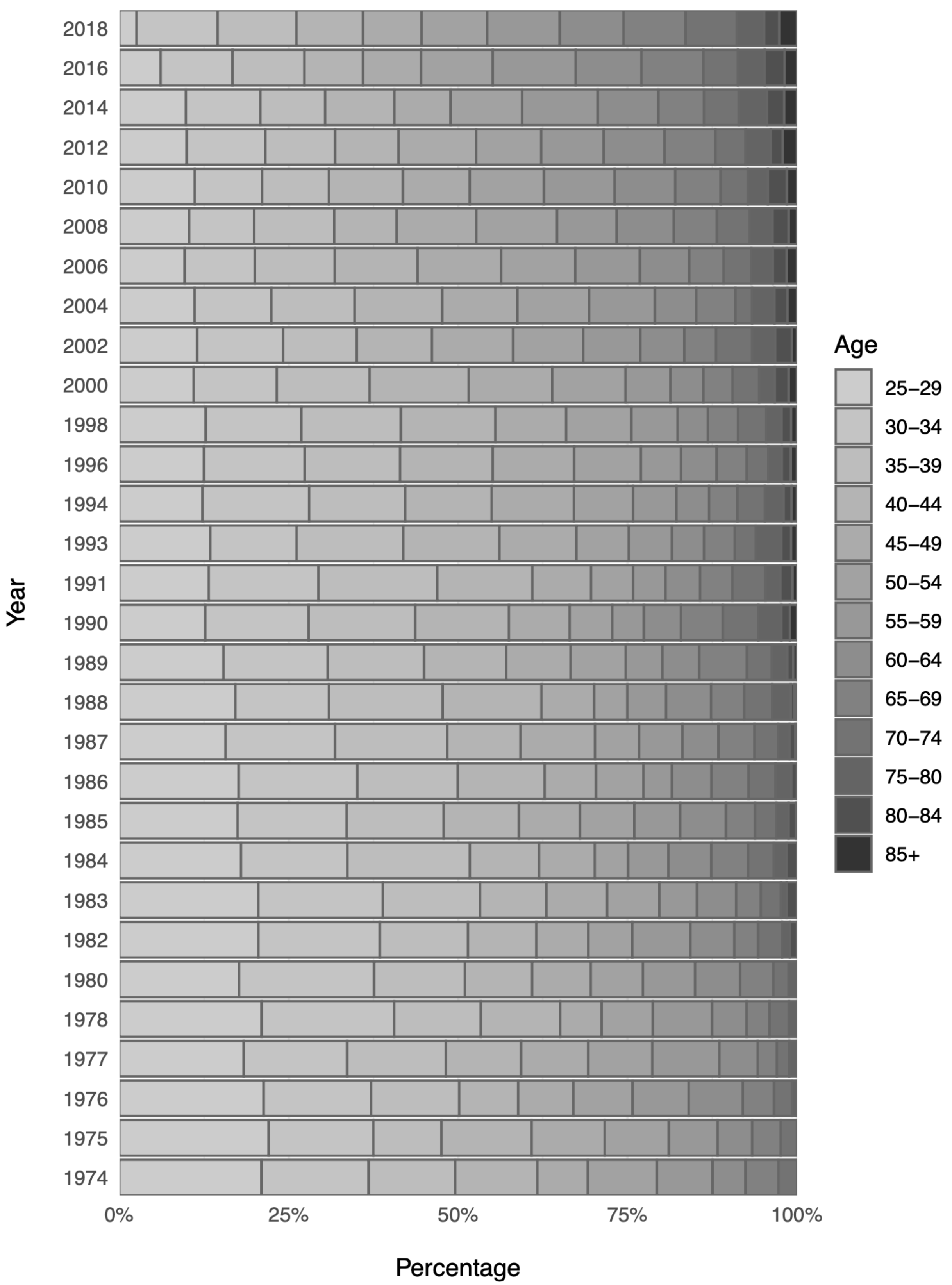

2018
2016
2014
2012
2010
2008
2006
2004
2002
2000
1998
1996
1994
1993
1991
1990
1989
1988
1987
1986
1985
1984
1983
1982
1980
1978
1977
1976
1975
1974
Year
0%
25%
50%
75%
100%
Percentage
Age
25–29
30–34
35–39
40–44
45–49
50–54
55–59
60–64
65–69
70–74
75–80
80–84
85+

Race

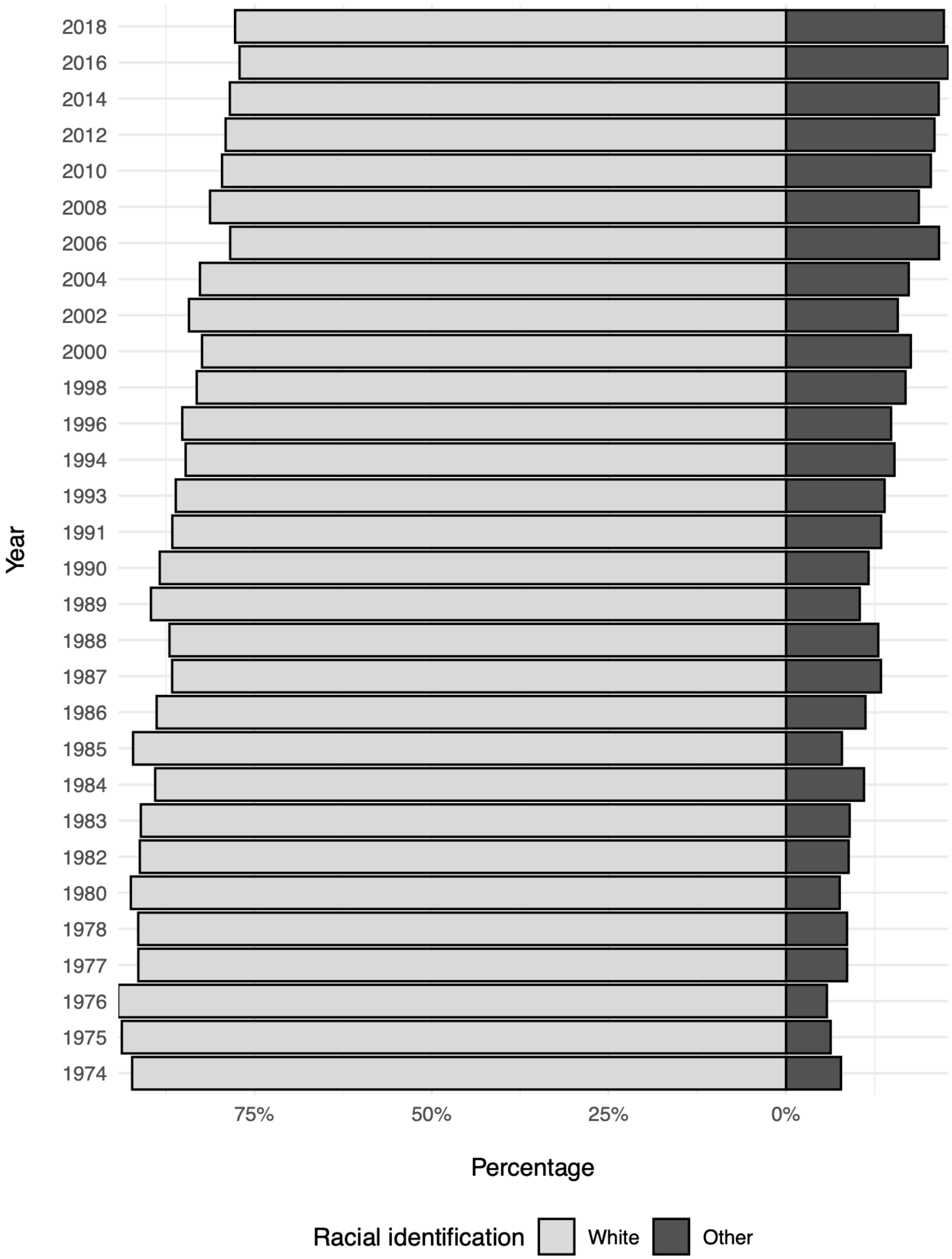
Year
2018
2016
2014
2012
2010
2008
2006
2004
2002
2000
1998
1996
1994
1993
1991
1990
1989
1988
1987
1986
1985
1984
1983
1982
1980
1978
1977
1976
1975
1974
75%
50%
25%
0%
Percentage
Racial identification
White
Other

Father's occupation (2010 census occupation codes, 10-fold)

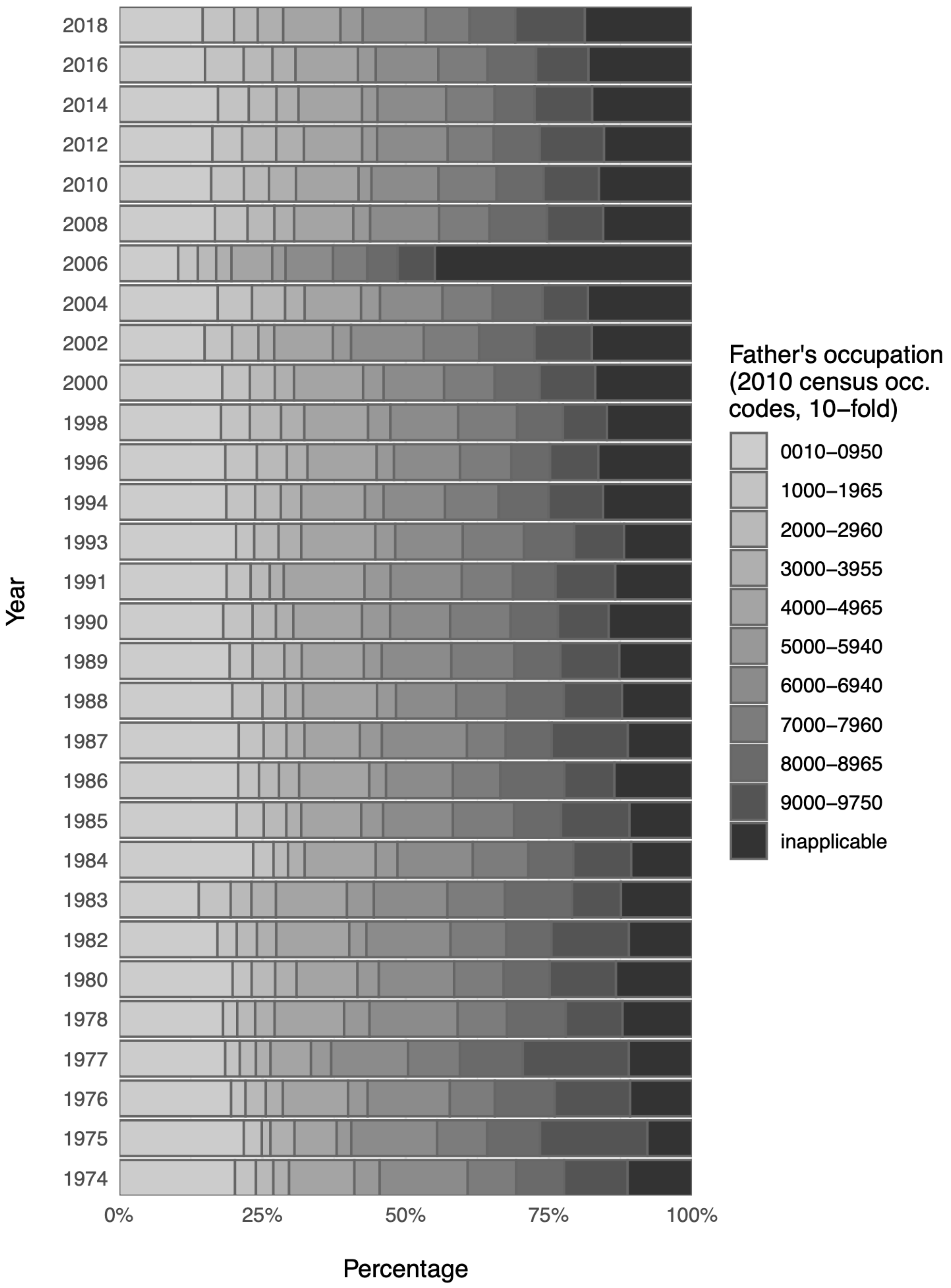

Father's occupational prestige

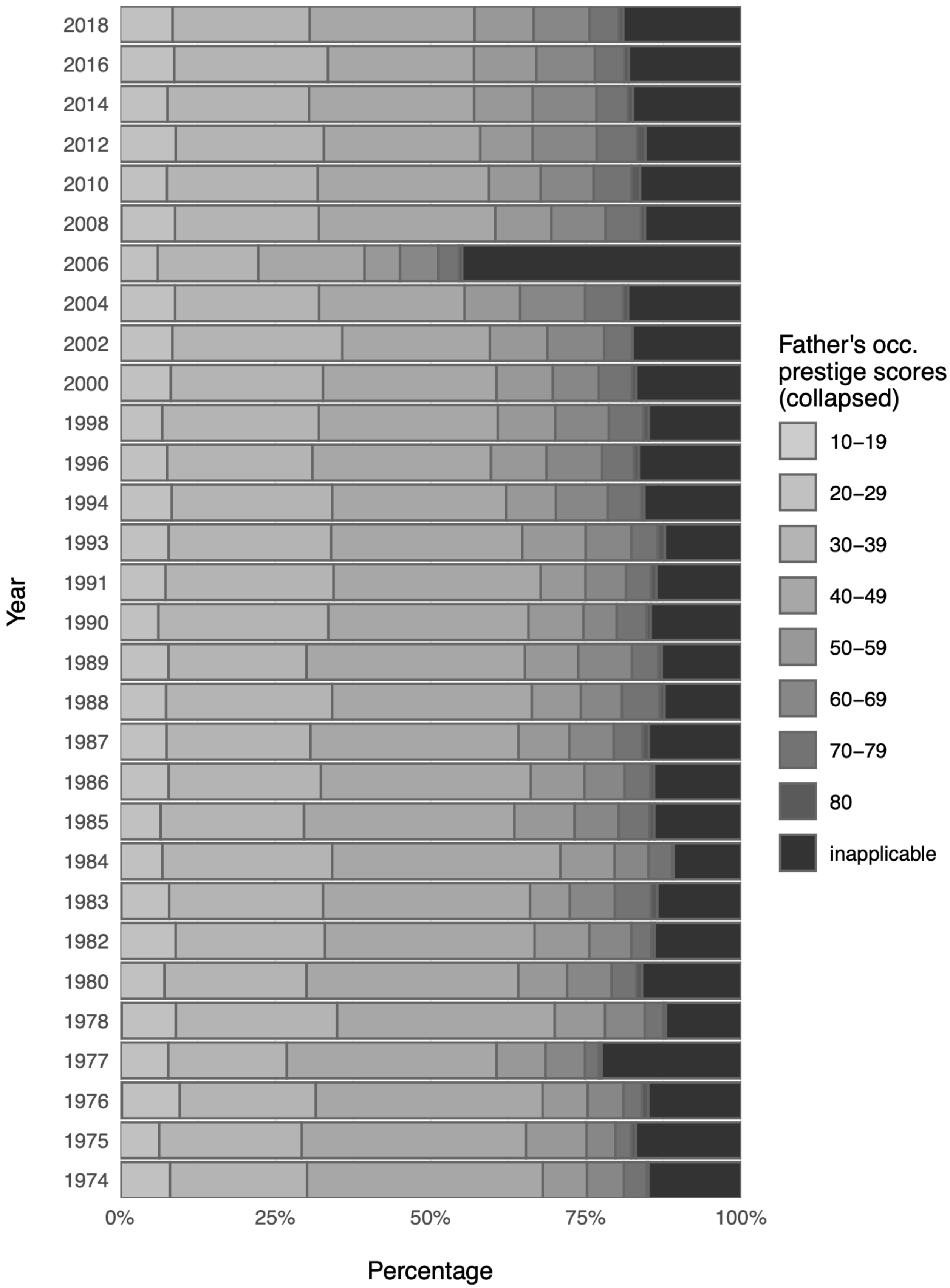

Father's employment status

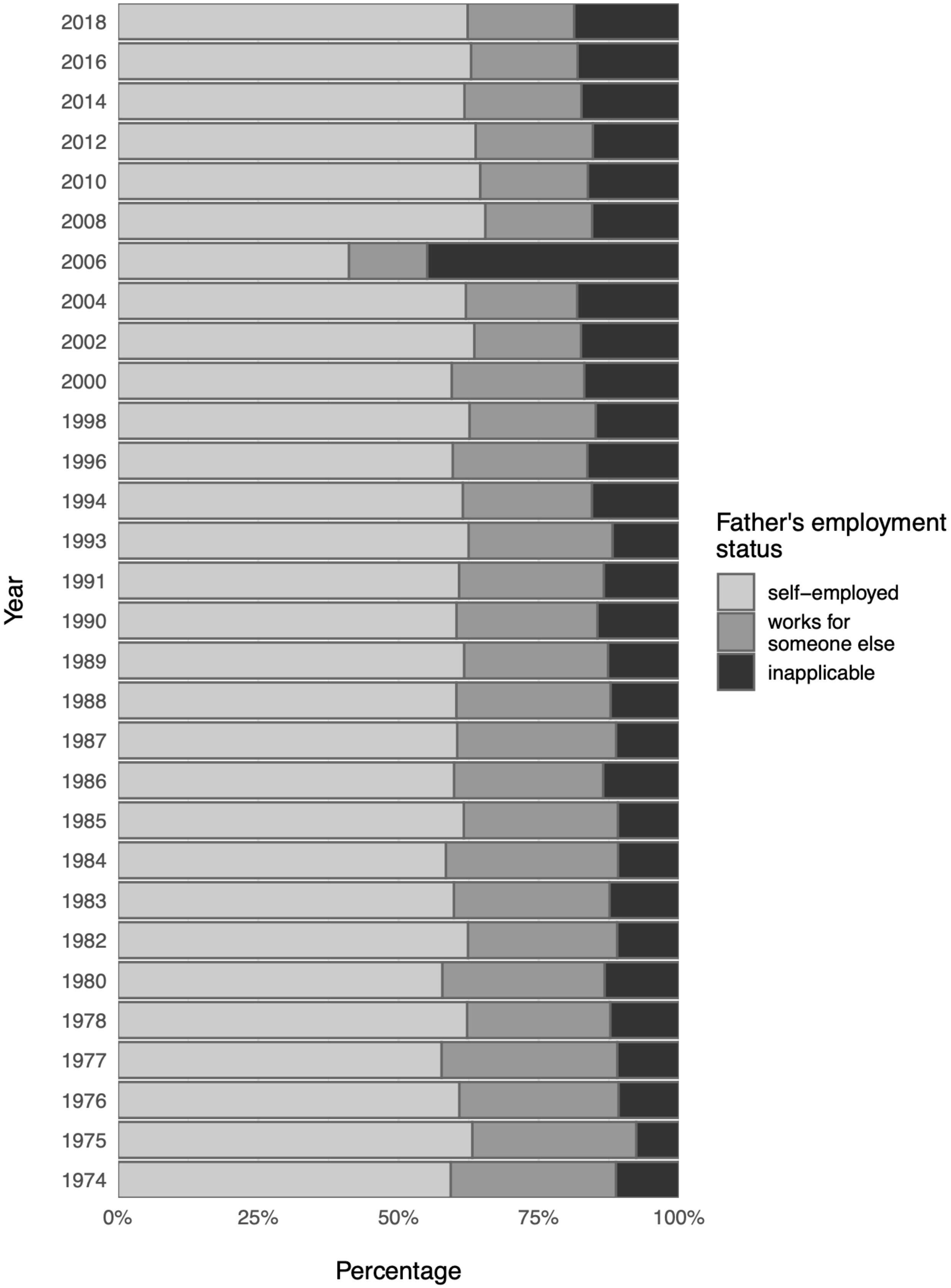

Father's highest educational degree

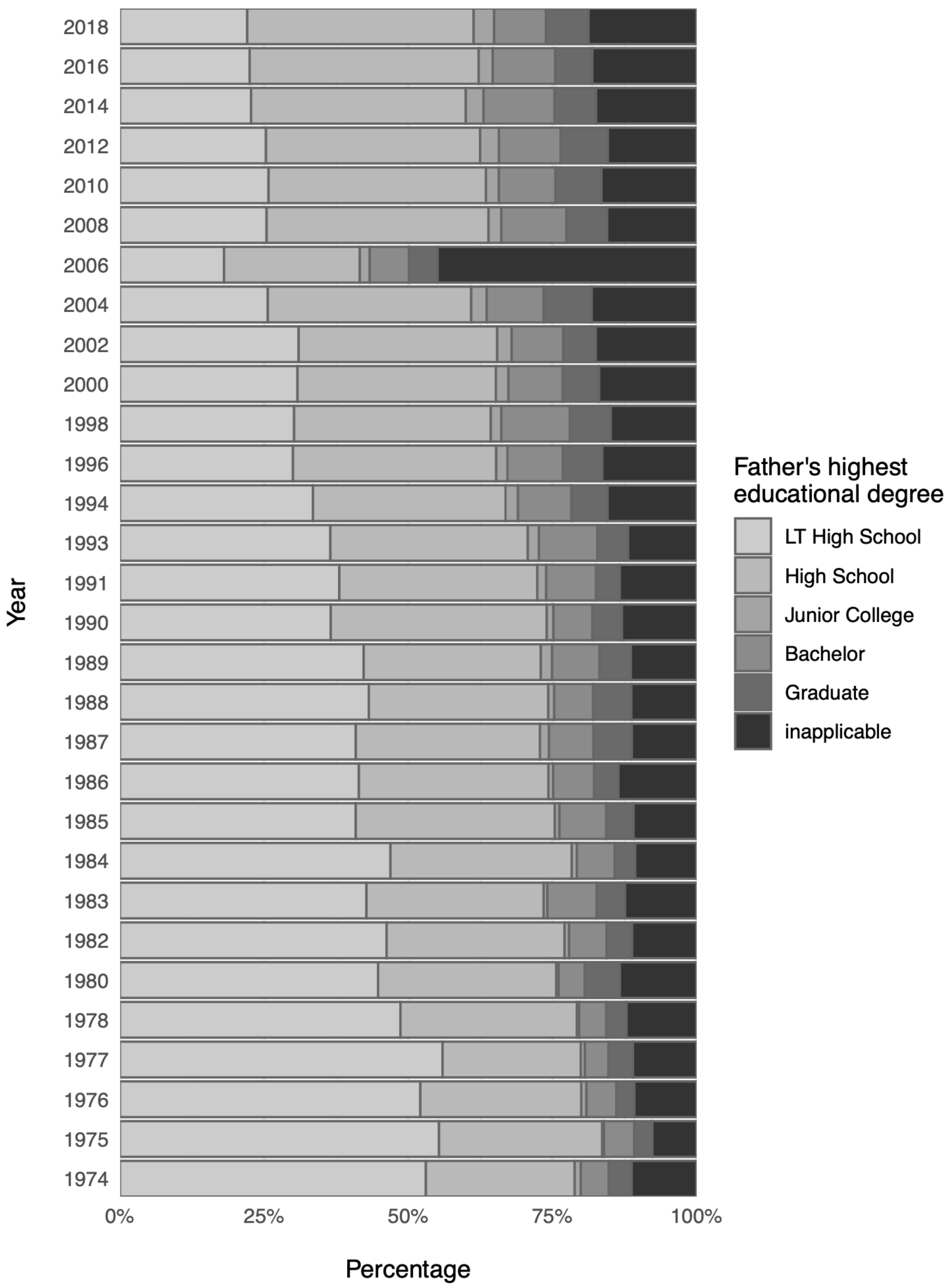

Mother's highest educational degree

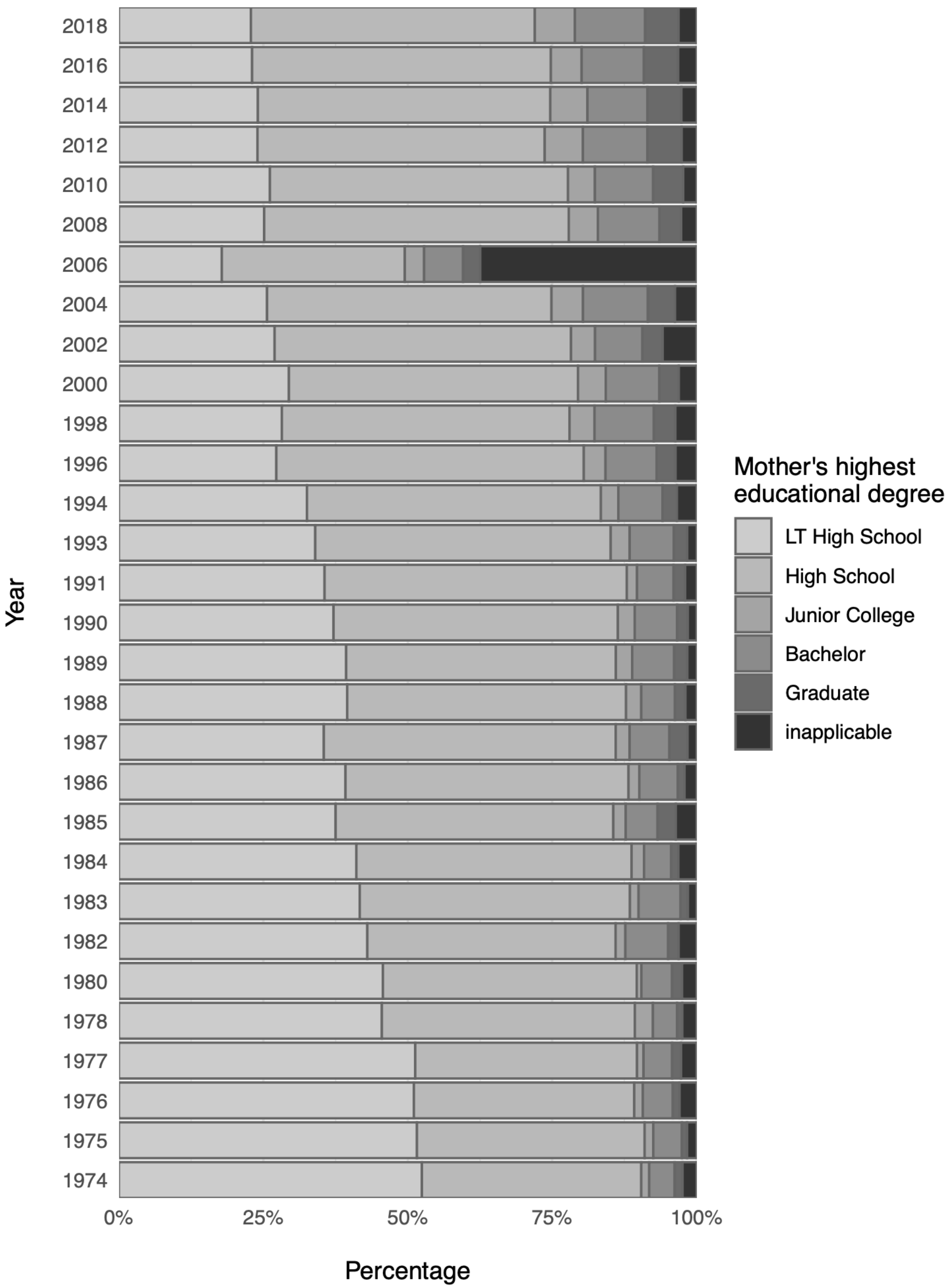

Family arrangement at age 16

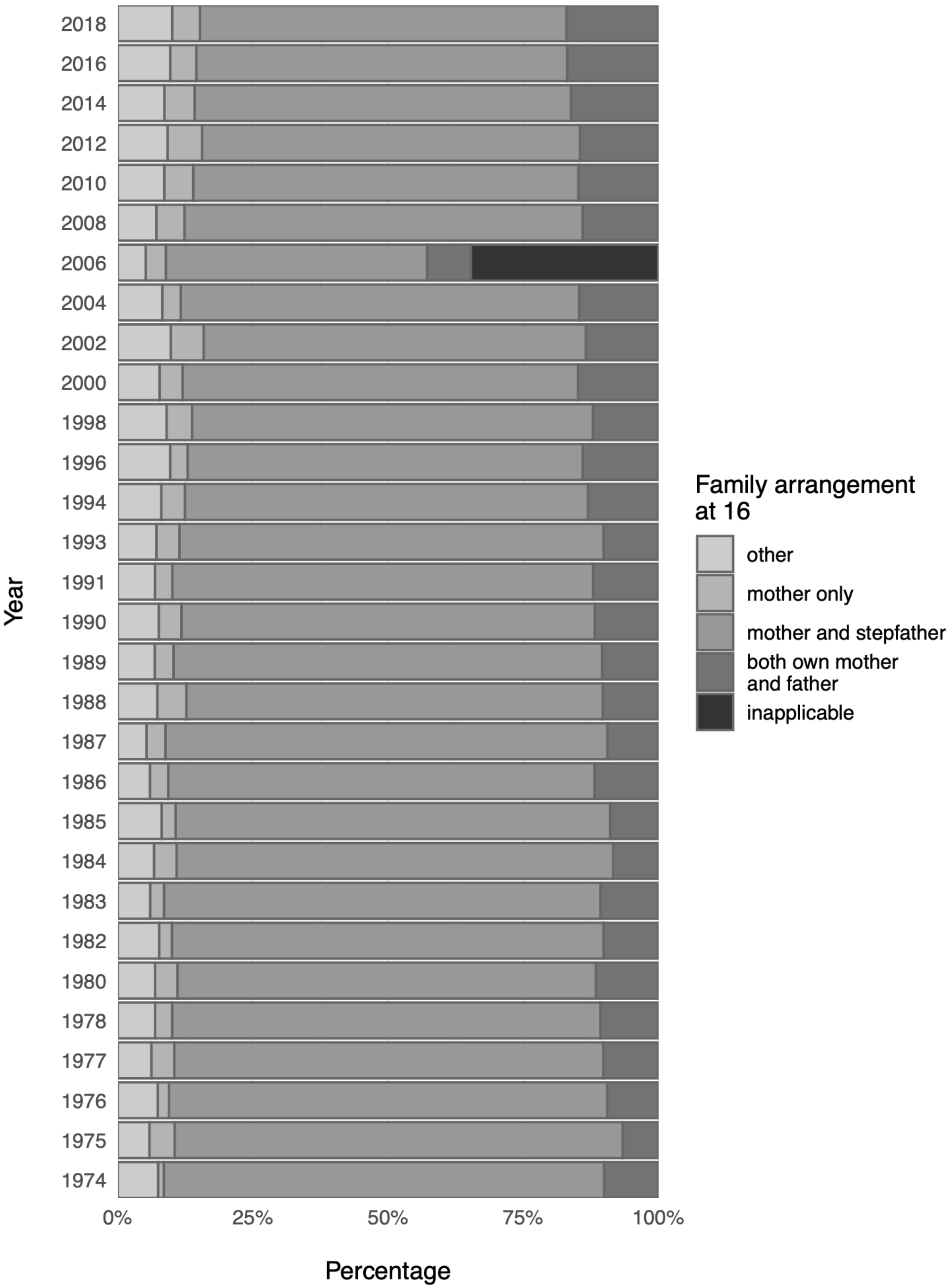

Religion raised in

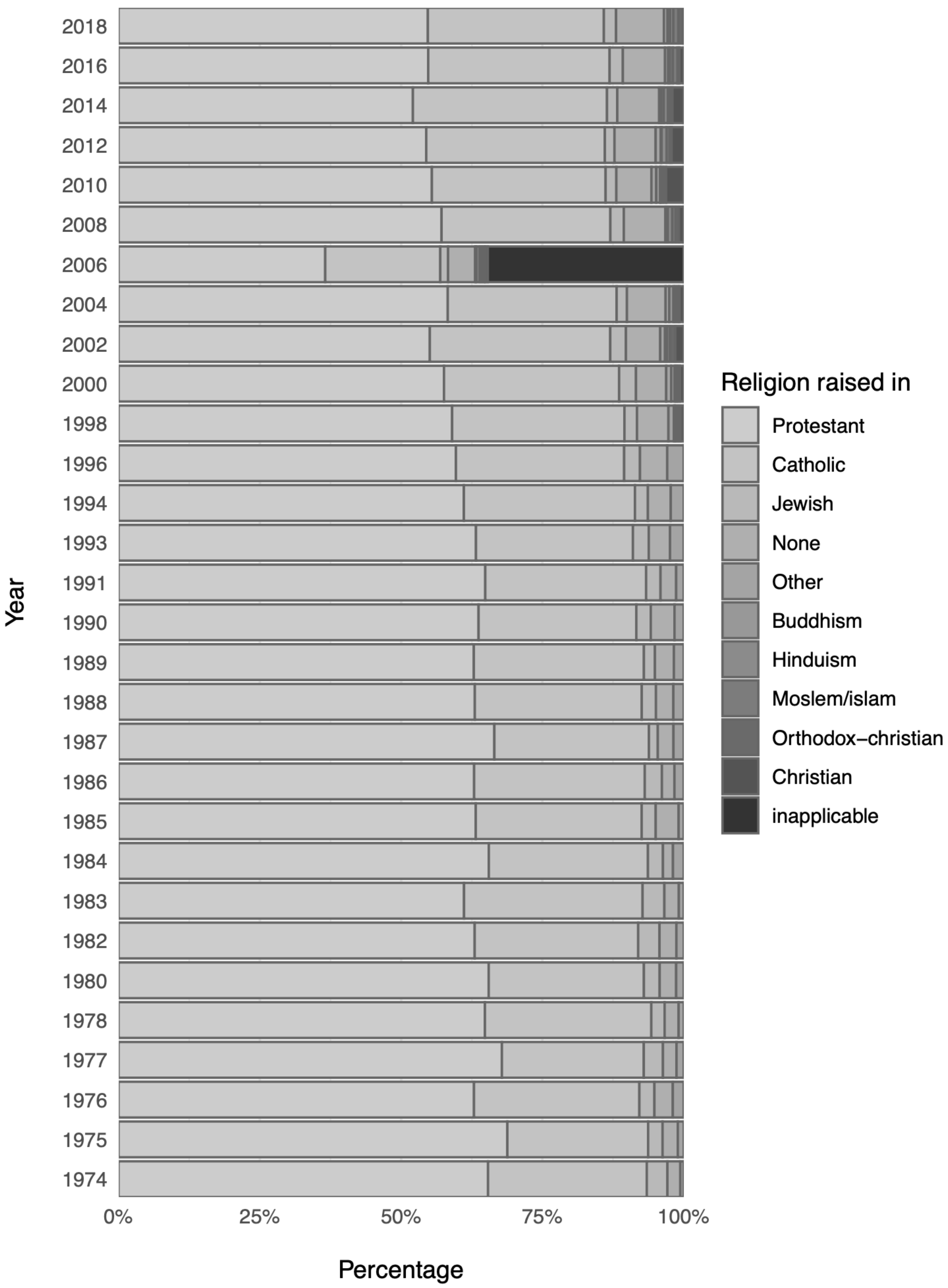

Denomination raised in

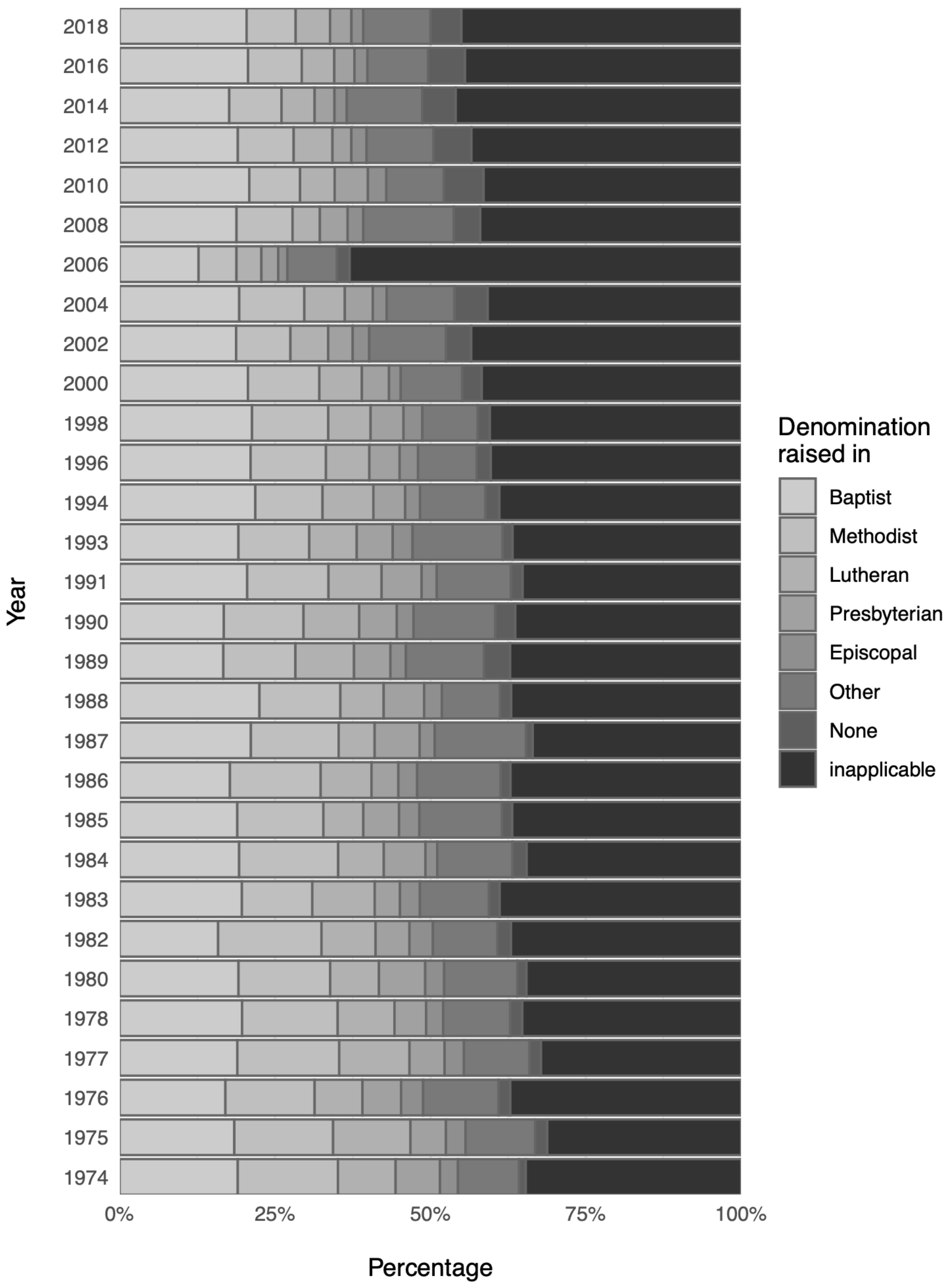

Type of place lived in at age 16

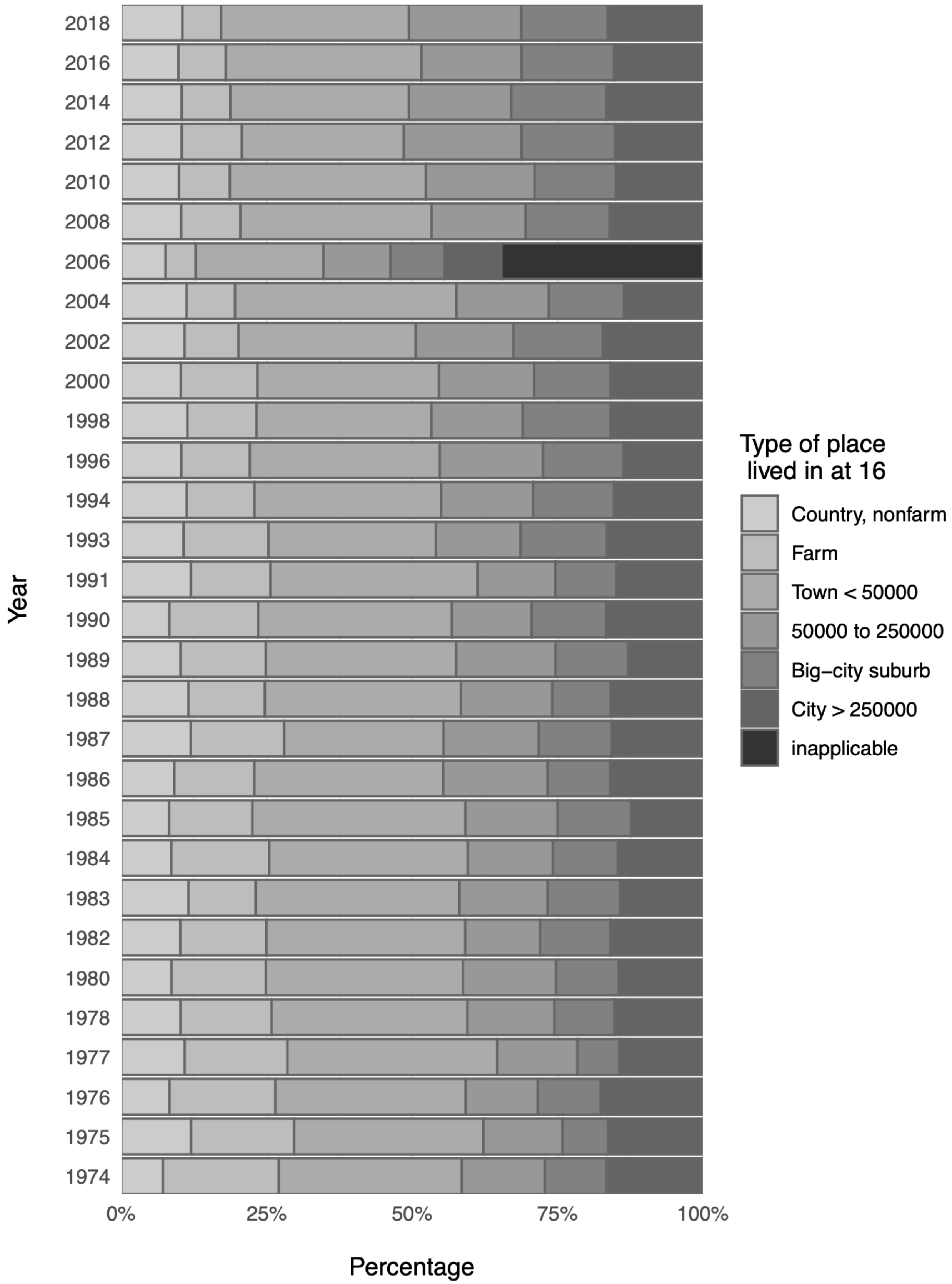

Region lived in at age 16

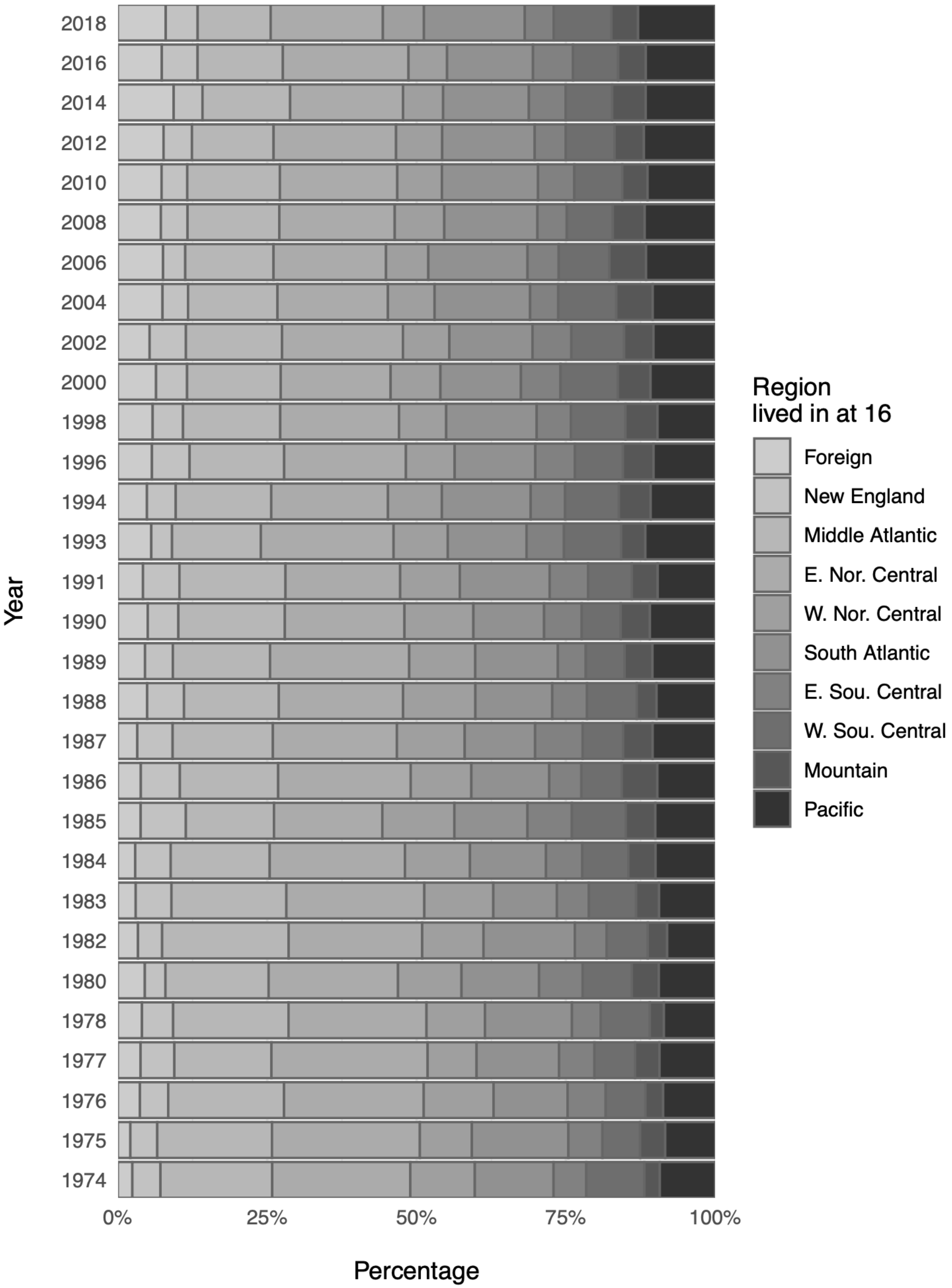

Supplement: S1 File — (PDF) [file pone.0279273.s003.pdf]
